# Emergence and Dissemination of *mcr*-Carrying Clinically Relevant *Salmonella* Typhimurium Monophasic Clone ST34

**DOI:** 10.3390/microorganisms7090298

**Published:** 2019-08-28

**Authors:** Silpak Biswas, Yan Li, Mohammed Elbediwi, Min Yue

**Affiliations:** 1 CATG Microbiology & Food Safety Laboratory, Institute of Preventive Veterinary Sciences, Zhejiang University College of Animal Sciences, Hangzhou 310058, China; 2 Zhejiang Provincial Key Laboratory of Preventive Veterinary Medicine, Hangzhou 310058, China

**Keywords:** Colistin, *mcr* gene, ST34, Multidrug resistance, Virulence

## Abstract

Antibiotic resistance in bacteria is one of the urgent threats to both public and global health. The *Salmonella* Typhimurium monophasic sequence type 34 (ST34) clone, with its rapid dissemination and resistance to numerous critical antimicrobials, has raised global concerns. Here, we present an updated overview on the emerging infections caused by mobile colistin resistance (*mcr*)-carrying colistin-resistant ST34 isolates, covering their global dissemination and virulence-associated efficacy. The higher rates of *mcr-1-*positive ST34 in children in China highlights the increasing threat caused by this pathogen. Most of the ST34 isolates carrying the *mcr-1* gene were isolated from animals and food products, indicating the role of foodborne transmission of *mcr-1*. The emergence of multidrug resistance genes along with various virulence factors and many heavy metal resistance genes on the chromosome and plasmid from ST34 isolates will challenge available therapeutic options. The presence of the colistin resistance gene (*mcr-1, mcr-3, and mcr-5*) with the multidrug-resistant phenotype in ST34 has spread across different countries, and most of the *mcr-1* genes in ST34 isolates were detected in plasmid type IncHI2 followed by IncI2, and IncX4. Together, *mcr-*carrying *S.* Typhimurium ST34 may become a new pandemic clone. The fast detection and active surveillance in community, hospital, animal herds, food products and environment are urgently warranted.

## 1. Introduction

*Salmonella enterica* serovars are one of the most common causative pathogens of enteric diseases in humans and animals over the world [1]. Non-typhoid *Salmonella* causes foodborne diseases, resulting in gastroenteritis diseases and sometimes bacteraemia. The ingestion of contaminated food, especially foods of animal origin, is believed to be the most common source for human infections [2]. The rate of antibiotic resistance (AR) and number of newly identified resistance determinants in *Salmonella* have increased significantly in the past decades [3,4,5]. Antibiotic-resistant *Salmonella* has become a major concern in the animal breeding section and in public health care system [6].

**Figure 1:**
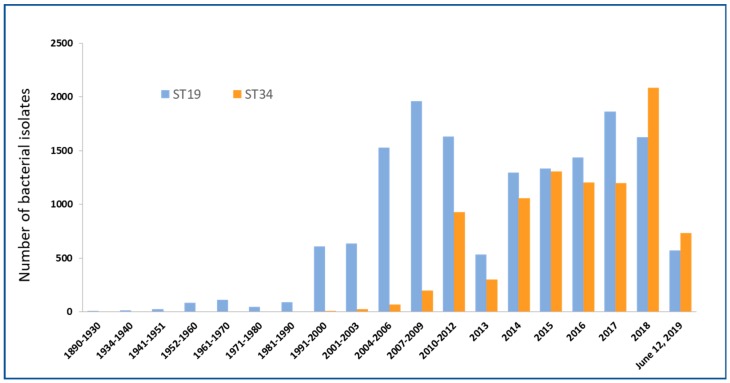
Emergence of global pandemic clone sequence type 34 (ST34) as compared to the “traditional” clone ST19 from 35,963 *Salmonella* Typhimurium isolates of the Enterobase [16]. Comparative analysis of number of isolates of ST19 and ST34 reported since 1890 till date shows that the increase in number of ST34 isolates in recent years.

**Figure 2:**
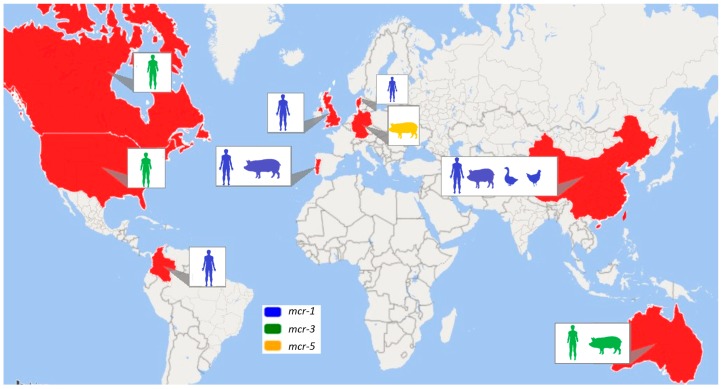
Global distribution of *Salmonella* Typhimurium ST34-carrying mobile colistin resistance (*mcr*)*-1, mcr-3*, and *mcr-5* colistin resistance genes [9,10,11,12,17,18,19,20,21,22,23,24,25,26]. This also shows the different sample sources from where *mcr-*carrying bacterial strains were obtained from different countries. In the non-red marked countries, no cases of *Salmonella* Typhimurium ST34-carrying *mcr* genes were reported.

*Salmonella enterica* serovar Typhimurium (*S.* Typhimurium) is one of the leading serovars responsible for global infectious diarrhoea and foodborne disease outbreak [7]. With time, several variants of *S.* Typhimurium, particularly a monophasic variant of Typhimurium, became dominant within animal and human samplings [8]. *S*. Typhimurium has also been defined using multilocus sequence typing (MLST), and it has been classified as sequence type or ST. ST19, ST34, ST313 and ST213 are commonly found STs of *S*. Typhimurium. Recently, one of the most prevalent *S.* Typhimurium has been ST34, with significant accumulation in the past two decades (Figure 1). The emergence of multidrug-resistant (MDR) monophasic ST34 has also been widely reported in Europe, North America, Asia, and Australia [9,10,11,12] (Figure 2). Food-producing animals are linked with human infections caused by *Salmonella* ST34 [13,14]. Importantly, ST34 is frequently associated with the ACSSuT (ampicillin, chloramphenicol, streptomycin, sulbactam, and tetracycline) resistance pattern, with an extension to critical antimicrobials [13]. The accumulation of MDR *S.* Typhimurium, including monophasic ST34, has significantly challenged current treatment options to control foodborne infections [15].

Colistin is a cationic polypeptide antibiotic which was discovered in the 1940s and showed significant antimicrobial activity against Gram-negative bacteria. The clinical use of colistin was started during the 1950s and has been used in veterinary and human medicine. Colistin use was greatly diminished during the 1980s due to its toxicity reported in clinical cases [27,28]. However, the increasing prevalence of MDR Gram-negative bacteria, along with the lack of new antibiotics against such strains, has led to a resurgence in colistin, and it has been recognized as the last-resort antibiotic for numerous MDR bacterial infections [27,29]. In the veterinary field, colistin is used more widely to treat infections caused by Gram-negative bacteria and more frequently as a growth promoter in livestock production [30,31]. Resistance to colistin found previously in bacteria is commonly due to chromosomal mutations in *pmrAB* and *phoPQ* which can confer the structural modification of lipid A [32,33,34]. Recently, the discovery of a plasmid-borne mobile colistin resistance (*mcr*) gene *mcr-1* challenged the use of ‘last-line’ antibiotic colistin in the treatment of bacterial infections, especially by MDR bacteria [31]. Mobile colistin resistance is known to be triggered by phosphoethanolamine (PEA) transferases [35,36,37]. Now, the existence of the *mcr* gene has been reported across different continents, demonstrating its importance as a newly recognized risk [38].

The MDR *S.* Typhimurium ST34 variant is a potential threat to public health due to its global expansion and the emergence of colistin resistance. Here, we highlight the prevalence, dissemination, and virulence properties of the rapidly emerging *mcr-*carrying multidrug-resistant monophasic clone *S.* Typhimurium ST34, which could pose significant pandemic risk.

## 2. Monophasic *Salmonella* Typhimurium

Most of the *Salmonella* serotypes are motile by flagella. An important characteristic of *Salmonella* is the existence of two different flagellin genes *fliC* and *fljB* on the bacterial chromosome. The expression of these two genes is done by a scheme named ‘phase variation.’ The majority of the *Salmonella* serotypes expresses both genes (also known as phase one and phase two) and is then called ‘biphasic.’ The *Salmonella* serotypes exhibiting only one flagellar phase are designated as ‘monophasic’ [39,40,41,42,43].

Monophasic *Salmonella* Typhimurium (mSTM), frequently linked with the ST34 lacking the *fljB*-encoded second phase H antigen, is one of the variants of *S.* Typhimurium [44,45]. There has been a rapid worldwide emergence of mSTM in the past two decades. In some European countries, mSTM ranks as the top *Salmonella* serovar among human and veterinary isolates, and it has become one of the most frequently characterized serovars from animals and food products [46]. Among various Typhimurium variants, ST34 (generally monophasic) and ST19 (generally biphasic) remain the top two.

The mSTM ST34 is circulating in multiple clonal lineages. Because of the rapid emergence of the clones with multidrug resistance properties, these variants are considered an emerging epidemic agent worldwide [47,48]. mSTM is the third most common serovar responsible for animal and human infections in European Union countries and is ranked among the top five in the USA [49,50]. Since 2009, it has been one of the most common serovars responsible for human salmonellosis in China. Pigs are considered one of the most significant vectors for mSTM ST34 [51,52].

## 3. Origin of *Salmonella* Typhimurium ST34

The observation of mSTM rising was started since 1990s. Its prevalence increased rapidly in Europe during the 2000s. Two major variants of mSTM have been reported, suggesting a convergent evolution. One is ST19, which is known as a ‘Spanish clone,’ and the other is ST34, which is acknowledged as a ‘European clone’ [53]. The mSTM European clone ST34 was found to be associated with resistant-type ASSuT (ampicillin, streptomycin, sulbactam, and tetracycline) [13]. On the other hand, resistant-type ACGSSuTTp (ampicillin, chloramphenicol, gentamicin, streptomycin/spectinomycin, sulfonamides, tetracyclines, and trimethoprim) was associated with the mSTM strains of the ‘Spanish clone’ ST19 [13]. From 35,963 *Salmonella* Typhimurium isolates collected from Enterobase [16] dataset, which includes ST19 and ST34, we observed that the global pandemic clone ST34 outcompetes or replaces the “traditional” clone ST19 (Figure 1). Table 1 illustrates the comparative analysis of the percentage of isolates of ST19 and ST34 using same Enterobase [16] dataset, which shows the increase in the percentage of ST34 isolates in recent years. It should be noted that majority of ST34 are monophasic, while a small percentage of ST19 show monophasic features.

The ‘European clone’ ST34 and the ‘Spanish clone’ ST19 emerged and spread worldwide, and they have been recognized as responsible for most human infections till date. The ‘Spanish clone’ (ST19) spread in Europe and the US (United States) after emerging in 1997 [54]. The ‘European clone’ or ST34 has become one of the major causes of non-typhoid *Salmonella* infections in humans in Europe in the 2000s [13]. The mSTM ST34 variant emerged in European countries in 2007 and then spread globally [55]. A genetic region of the ST34 clone containing the *fljAB-hin* operon has been replaced by a composite transposon insertion in the chromosome which contains antibiotic resistance genes such as *strA*, *strB*, *sul2*, *tet*(B), and *bla*_TEM-1_ [53]. The ‘European clone’ has been most commonly reported in numerous countries of Europe [9,17], but also in America [56], Asia [53], and Australia [11].

## 4. Global Distribution of *mcr-1*-Carrying Pandemic Clone ST34

Mobilized colistin resistance is evolving very rapidly in an array of bacteria, and its global dissemination poses a great threat to human health. The animal or human hosts origin, as well as the hosting plasmids, in the mSTM ST34 clone are ongoing active fields with heavy attention. Table 2 shows the characteristics of the *mcr-*positive *Salmonella* Typhimurium ST34 found in different studies worldwide. Figure 2 shows global distribution of colistin-resistant *Salmonella* Typhimurium ST34-carrying *mcr-1, mcr-3*, and *mcr-5* genes.

### 4.1. mcr-1-Positive ST34 Isolates from Human

Diarrhoea is one of the leading causes of mortality in children under five years of age [57]. *Salmonella* spp. are responsible for causing bacterial diarrhoea in children aged under five years of age. A Chinese group detected *mcr-1* gene occurrence in *Salmonella* strains collected from diarrhoeal outpatients aged under five years in Shanghai during 2006–2016 [18]. All *mcr-1*-harbouring *Salmonella* strains were reported to be carrying MDR plasmids, with resistance to different clinically important antibiotics. These data indicate that the bacterial infection of children with *mcr-1*-mediated colistin resistance has become a significant emerging antibiotic resistance problem. Interestingly, most *mcr-1*-positive mSTM ST34 strains were clustered together with the pork strains, which strongly suggests pork eating as a main bacterial infection source. Another Chinese group found that three *mcr-1* harbouring colistin-resistant ST34 have been isolated from the faeces of patients with diarrhoea in Zhejiang Province, China between 2007 and 2016 [19]. All three isolates carried various antibiotic resistance genes with MDR features. The *mcr-1* positive ST34 isolates were resistant to third and fourth-generation cephalosporins and trimethoprim-sulfamethoxazole in addition to colistin.

In 2017, the presence of *mcr-1* in Colombian clinical isolates of ST34 were also reported, and these ST34 isolates showed a common MDR phenotype, including tetracycline, ampicillin, nalidixic acid, and colistin, with some resistant to chloramphenicol, cephalothin, cefoxitin and ciprofloxacin [20]. Three common AR genes were identified, including *bla*_TEM-1_, *qnrB19*, and *tetB* [20] (Table 3). A novel *mcr-1.6* variant was found in a colistin-resistant *Salmonella* Typhimurium ST34 strain from a healthy person from China. The strain-carrying *mcr-1.6* gene showed an MDR phenotype and carried several resistance genes [21]. These studies have highlighted mSTM as one of the significant pathogens for community-acquired and hospital-associated infections.

### 4.2. mcr-1-Positive ST34 Isolates from Pig and Its Products

In 2016, the *mcr-1* gene was found in MDR and copper-tolerant *Salmonella* spp. from pigs in Portugal [17]. Interestingly, the *mcr-1*-positive *Salmonella* isolates were associated with particular MDR clones such as mSTM ST34, and they were found during 2002–2015. This is most likely due to high consumption of polymyxins in the Portugal swine industry in the past decades [58,59].

One *mcr-1* positive mSTM ST34 obtained from ready-to-eat pork (RTE) samples was reported in China in 2014 [22]. Twenty-one AR genes and 201 virulence factors were identified on the chromosome of the mSTM ST34 strain. Recently, the detection of the *mcr-1* gene in *Salmonella* isolates from pigs at slaughterhouse found twenty-one (14.8%) strains, including nineteen ST34 [23]. It was also indicated that spread of the *mcr-1* gene in pigs at a slaughterhouse in China was associated with the clonal distribution of mSTM ST34 variants. Collectively, pork consumption is a possible source of the major contamination of colistin-resistant ST34 strains. This suggests that the spread of this ST34 clone would act as an emerging public health risk via efficient food-chain migration.

### 4.3. mcr-1-Positive ST34 Isolates from Other Animals

Poultry, including chicken, duck and turkey, also plays an important role in hosting the *mcr-1*-positive ST34 clone. 22 *S. enterica* isolates (out of 276 isolates obtained from duck and chicken in China) were colistin-resistant [10]. Among these, only five isolates belonged to ST34 *S.* Typhimurium were found as *mcr-1* positive isolates. The dissemination of the *mcr-1* gene was found through the clonal spread of plasmid-mediated quinolone resistance carrying ST34 isolates. All the *mcr-1* positive *S.* Typhimurium ST34 exhibited MDR phenotypes, including colistin, gentamicin, ampicillin, streptomycin, tetracycline, florfenicol, trimethoprim-sulfamethoxazole, and nalidixic acid (Table 3). Interestingly, all the *mcr-1* positive *Salmonella* ST34 isolates were obtained from diseased animals. On the other hand, most of the colistin-resistant ST34 strains which were not carrying the *mcr-1* gene were from healthy animals [10].

### 4.4. Diversified mcr-1 Carrying Plasmids in ST34

The *mcr* genes are generally hosted on the bacterial plasmids that are highly mobile and this accelerate the spread of resistance under the selection pressure [60]. The *mcr* genes have been detected in a wide range of plasmid types including IncI2, IncHI2, and IncX4 in numerous studies [61]. The examined ST34 isolates in Portugal with the *mcr-1* gene, carried by IncX4 and IncHI2 plasmids, showed a minimum inhibitory concentration (MIC) of 4–8 mg/L to colistin [17]. All the *mcr-1* plasmids were found transferable, and it has also been suggested that *S*. Typhimurium acquired the *mcr-1* plasmids easier as compared to *S*. Enteritidis, which is another common serotype of *Salmonella* [18]. In 2017, a novel *mcr-1.6*, which is a variant of the *mcr-1* gene, was found in an IncP plasmid in a colistin-resistant ST34 strain from a healthy person in China [21]. A UK group identified fifteen *mcr-1*-positive isolates by the rapid screening of ~24,000 genomes of different bacteria, including *Salmonella* isolated from humans or food [9]. Among fifteen *mcr-1*-positive isolates, ten were *Salmonella* isolates and four belonged to ST34. The *mcr-1* gene was detected on different plasmids, including IncHI2, IncI2 and IncX4 [9]. A single plasmid was identified and found to be an IncHI2/HI2A type which encoded an *mcr-1* gene in one mSTM ST34 isolate obtained from RTE [22]. The *mcr-1* gene in *Salmonella* ST34 isolates from pigs in China was located mainly on IncHI2-like plasmids [23]. In another study, three *mcr-1* harbouring colistin-resistant ST34 were isolated from a diarrhoea patient in China, with one in IncI2 and two in IncHI2 [19].

Here, we found that most of the *mcr-1* genes in ST34 isolates were detected in plasmid type IncHI2 followed by IncI2 and IncX4 (Table 2). IncHI2 plasmids are well-known for their ability to transfer by conjugation in a wide range of temperatures. Several genes, encoding for antitoxin systems, colicin, tellurite, heavy metals, and AR genes likely play critical roles in the stability of IncHI2 plasmids in the ST34 clone [62].

## 5. MDR and *mcr-3*-Carrying ST34 Linked with International Travel

*mcr-3*, a novel *mcr* variant, was discovered on a conjugative plasmid from *E. coli* of pig origin in China in 2017 [63]. The *mcr-3* gene was found in a colistin-resistant isolate obtained from a faecal sample of an apparently healthy pig in Shandong, China, in 2015. This *mcr-3-*carrying isolate also possessed many AR genes. Since it was first identified in Shandong, the *mcr-3* has been found in several countries in MDR bacterial infections [64]. The mSTM ST34 containing *mcr-3* was identified in Canada recently, and the patient had travelled to Thailand, linked with foodborne transmission [24] (Table 2). Interestingly, another study reported that patients with mSTM infections carrying *mcr-3* also had travel histories to Thailand and Vietnam [25]. *mcr-3*-carrying ST34 strains were reported from stool, blood or urine samples from patients in Denmark during 2009–2017 (Figure 2). Ten *Salmonella* isolates were found *mcr-3-*positive, and, interestingly, one isolate was carrying both the *mcr-1* gene and the *mcr-3* gene. The level of AR in the Danish human cases of *S.* Typhimurium and the mSTM variant is higher, particularly in cases with travel history [25]. A retrospective study found 54 mSTM isolates from humans and two isolates from pork meat from Australia (Table 2). All mSTM isolates were ST34, and *mcr-3* was identified in MDR *Salmonella* ST34 from an Australian resident who had travelled to Vietnam [11]. The colistin-resistant mSTM ST34 harbouring *mcr-3.1* was also recovered very recently from a patient from the USA who travelled to China two weeks prior to diarrhoea [12]. Together, these reports showed that the unique transmission pattern in MDR ST34-carrying *mcr-3* and could indicate Southeast Asian as the potential reservoir for the global dissemination of *mcr-3*-containing ST34.

## 6. Novel *mcr-5*-Harbouring ST34

In 2017, a German group firstly reported the *mcr-5* gene in *Salmonella* Paratyphi B from poultry and food [65]. The *mcr-5* positive *Salmonella* Typhimurium ST34 isolates were obtained from pig and meat in Germany and reported very recently [26] (Figure 2). A PCR-screening of 315 colistin-resistant *Salmonella* isolates revealed that *mcr-5* was harboured by eight *Salmonella* strains in five German federal states. MIC testing results confirmed that *mcr-5* location seems to have a major effect on the MIC value. Five plasmid types, including three novel types, were detected to be harbouring the *mcr-5* gene in mSTM isolates (Table 2).

## 7. Virulence Associated Features in mSTM ST34

### 7.1. Resilience to Heavy Metal

Copper and zinc supplementation are commonly-used ways to enhance animal growth, including in the swine industry, while limiting antibiotic usage. Accordingly, heavy metals could accumulate and persist in soil, water, and sediments, leading to a selection of bacteria with heavy metal resistance [66]. The mSTM ST34 variant is primarily associated with pigs with copper resistance. Furthermore, this heavy metal pressures in the pig setting environment could contribute to the co-selection of MDR clones, which ultimately affects food safety and human health [67,68,69]. One *mcr-1* positive mSTM ST34 obtained from pork, reported in China in 2014, was carrying twenty-eight resistance genes associated with different metals, i.e., copper and mercury [22] (Table 3).

A previous study demonstrated that the acquisition of copper and silver tolerance genes could contribute to the emergence of MDR *Salmonella* serotypes in pig production [68]. Historically, the copper and silver resistance genes were rarely associated with *Salmonella*, while they were significantly associated with lineages of mSTM, including the European clone and the Spanish clone [55]. While commonly found on the chromosome of the mSTM ST34 clone, IncHI2 plasmids have been found frequently associated with various metal tolerance genes such as the *sil*/*pco* genes [70,71]. Recent findings have suggested genomic island-3 and -4 are linked with copper tolerance [72,73]. These evidences support the rapid transmission of heavy metal resistance in piggery-related settings.

### 7.2. Biofilm-Forming Abilities

A recent study demonstrated the strong biofilm-forming ability of MDR *S*. Typhimurium ST34 from patients in Chinese southern coastal regions [74]. It has been reported previously that the ability to form biofilms by foodborne pathogens was significantly related to human diseases and the increased risk of severe outcomes [75]. *S*. Typhimurium ST34 was the most common genotype, followed by ST19, showing strong biofilm abilities and a higher MDR rate when compared with ST19 [74]. This incident was also seen in some previous studies [3,76]. This trend was similar regarding biofilm production ability to create improved bacterial fitness to an unfavourable environment, including heavy metals and antimicrobial resistance, by promoting the evolution of the MDR phenotype.

### 7.3. Virulence Potential in Cellular and Animal Model

Several studies have conducted *in vitro* and *in vivo* infection assays to evaluate the virulence potential of this emerging clone. Since pig or pork were considered the major reservoirs for mSTM ST34, several independent studies, using the porcine intestinal cell line (IPEC-1) and a specific-pathogen-free piglet infection model, found no significant difference between classic biphasic ST19 and the newly predominant monophasic ST34 clone in terms of colonization, serology response, and bacterial shedding [77,78,79], and a recent study suggested a significant enteric disease burden in swine population by showing pathological evidence [80]. An additional study used a chick infection model to assess colonization and virulence features, while no significant difference was detected between ST19 and ST34 [81,82]. However, an earlier study suggested that mSTM, isolated from wild birds, showed a highly invasive and killing feature [83]. Interestingly, all the wild bird isolates were pan-susceptible to eleven commonly used antimicrobials. The pathogenesis of mSTM ST34, compared to classic ST19, is likely due to the patho-adaptive evolution by allelic variations and horizontal gene transfer events, which are worthy of further investigation [84,85,86,87].

## 8. Conclusions

This review outlines some essential and updated knowledge about the prevalence and dissemination, as well as the virulence properties, of rapidly emerging *mcr-*carrying and MDR mSTM ST34. The limitation of this review is that only the references with “ST34” have been included here due to the lack of MLST data in some studies, which only reported monophasic variants by serological study but did not report the sequence type. The global dissemination of the MDR ST34 clone is likely an emerging threat to both global and public health, and, as such, it warrants being closely monitored in different sectors, including community populations and hospital patients, as well as animal herds, their food products, and their environments [88]. The *mcr* gene spreading mediated by IncHI2-like plasmids, along with ST34 variants, highlights the necessity to understand the genetic mechanism of bacteria–plasmid pairs. The detection of *mcr-1* in copper-tolerant clones challenges the efficacy of recently suggested metal-based interventions, i.e., copper, to reduce the use of colistin and *mcr-1* dissemination. Improved strategies are required to slow down the transmission in both clinical settings and environments in the context of the global dissemination of the MDR ST34 clone.

Further global phylo-genomics studies and ecological investigations will provide critical knowledge about the origin and evolution of the life-threatening mSTM ST34 and will improve understanding in the development of MDR (including *mcr*), enhancement virulence, and host preference. To prevent the overuse and misuse of colistin, the recommendation of use of this antibiotic needs to be strengthened both nationally and internationally. An integrated global one-health approach will be of significant importance to reduce unnecessary colistin use and reduce the further spread of *mcr*-carrying microorganisms and associated infections in both the veterinary field and the public health care system.

## Figures and Tables

**Table 1:** A comparative analysis of the percentage (%) of isolates of ST19 and ST34 from 35,963 *Salmonella* Typhimurium isolates of the Enterobase [16] shows the increase in the % of ST34 isolates in recent years.

Years	% ST19 isolates	% ST34 Isolates
**1890–1930**	100	0
**1934–1940**	100	0
**1941–1951**	100	0
**1952–1960**	100	0
**1961–1970**	100	0
**1971–1980**	100	0
**1981–1990**	100	0
**1991–2000**	98.38449	1.615509
**2001–2003**	96.35258	3.647416
**2004–2006**	95.56804	4.43196
**2007–2009**	90.86271	9.137291
**2010–2012**	63.74658	36.25342
**2013**	64.14868	35.85132
**2014**	55.09771	44.90229
**2015**	50.5104	49.4896
**2016**	54.48694	45.51306
**2017**	60.84259	39.15741
**2018**	43.78029	56.21971
**June 12, 2019**	43.76435	56.23565

**Table 2:** Characteristics of the *mcr-*positive *Salmonella* Typhimurium monophasic clone ST34 found in different studies worldwide.

Year of Publication	Country	Year of Sampling	*mcr* type	Colistin MIC	Host	Sample Source	Sample Type	No. of Samples	No. of *Salmonella* Isolates	No of *mcr* Positive ST34	Plasmid Type Detected	Comments	Travel History
2016	China [10]	2007–2015	*mcr-1*	16 mg/L	Animals	Duck, chicken, swine	Faecal	276	22	5	IncI2, IncHI2		
2016	England and Wales [9]	2012–2015	*mcr-1*	4–8 mg/L	Human	Human	Faeces	>24，000	17，684	4	IncX4, IncHI2		Thailand, Cambodia
2016	Portugal [17]	2002–2015	*mcr-1*	4–8 mg/L	Human and Animals		Faeces/blood, Pork meat/carcass	1010	1010	11	IncHI2, IncX4	Carried metal tolerance genes, (carrying *pcoD + silA* on the chromosome)	
2017	Denmark [25]	2009–2017	*mcr-1* and *mcr-3*		Human	Human	Stool, blood or urine	2500		10 [1 (*mcr-1* + *mcr-3*); and 9 (*mcr-3*)]	IncHI2A, IncHI2, IncN, TrfA, IncQ1, ColRNAI, IncA/C2, IncFII, IncX1, IncFIC(FII), IncI2		Four of the patients had travelled to Thailand and one to Vietnam before onset of disease; three patients had unknown travel history.
2017	China [23]	2013–2014	*mcr-1*	1–2 mg/L	Animals	Pigs	Cecum	1780	142	19	IncHI2		
2017	China [21]	2014	*mcr-1.6*	4 mg/L	Human	Human	Rectal swab		1	1	IncP		
2017	Columbia [20]	2012–2016	*mcr-1*		Human	Human	Stool, urine	5887	13	3	ColpVC, IncQ1, IncFIA, IncHI1A, IncHI1B		
2018	Australia [11]	2016–2017	*mcr-3*	4 mg/L	Humans and animals	Human, pigs		971	80	56			The isolate from the case-patient who travelled to Vietnam
2018	Canada [24]	2013	*mcr-3.2*	>4 mg/L	Human	Human	Faecal		1	1	IncHI2		Man with previous travel history to Thailand
2018	China [22]	2014	*mcr-1*	8 mg/L	Animals	Pigs	Prepared pork that is ready-to-eat	3200	30	1	IncHI2, IncHI2A		
2019	China [19]	2007– 2016	*mcr-1*	8 mg/L	Human	Human	Faeces	62	62	3	IncI2, IncHI2	Patients with infectious diarrhoea. 2 were infants and the other was 15 years old.	
2019	China [18]	2006–2016	*mcr-1*	4–8 mg/L	Human	Human	Faeces	134868	12053	37	IncHI2, IncI2, IncX4	Among the 37 patients infected with *mcr-1*-positive *Salmonella*, 33 (89%) were aged under 5 years.	
2019	Germany [26]		*mcr-5*	4 mg/L	animals	pigs	Faeces and meat		315	8	ColE-like, IncX1		
2019	USA [12]	2014–2016	*mcr-3.1*	>4 mg/L	Human	Human	Faecal		100	1	IncHI2		18 years old man with previous travel history to China

**Table 3:** Antibiotic resistance pattern, antibiotic resistance genes, virulence resistance-encoding genes and metal resistance genes found in *mcr-*positive ST34 isolates in different studies. NA = Not available.

References	*mcr* Gene Found	Antibiotic Resistance Phenotype	Antibiotic Resistance Genes	Virulence Resistance-Encoding Genes	Metal Resistance Genes
[9]	*mcr-1*	Colistin, β-Lactam, fluoroquinolone	*bla*_TEM-1_, *qnrS1*	NA	NA
[17]	*mcr-1*	Colistin, ampicillin, gentamicin, streptomycin, sulfamethoxazole; tetracycline; chloramphenicol; ciprofloxacin; pefloxacin, trimethoprim	*bla*_TEM_, *aac(3)-IV*, *strA-strB*, *sul2*, *tet*(B), *strA-strB*, *sul2*, *floR, catA-cmlA*, *aadA1/aadA2*, *sul1-sul3/sul2*, *tet*(A), *dfrA1/dfrA12*	NA	*pcoD* + *silA + merA* + *terF*
[10]	*mcr-1*	Colistin, nalidixic acid, olaquindox, ampicillin, streptomycin, gentamicin, florfenicol, tetracycline, trimethoprim-sulfamethoxazole	*oqxAB*, *aac*(*6*′)*-Ib-cr, floR,*	NA	NA
[23]	*mcr-1*	Colistin, ampicillin, streptomycin, florfenicol, tetracycline, sulfamethoxazole/trimethoprim, gentamicin	*floR*, *oqxAB*	NA	NA
[20]	*mcr-1*	Colistin, tetracycline, ampicillin, nalidixic acid, colistin, chloramphenicol, cephalothin, and cefoxitin, ciprofloxacin	*bla*_TEM-1_, *qnrB19*, *strA*, *strB*, *sul2*, *tetB, floR*, *tetA, aph*(*3″*)-*Ib, aph*(*6*)-*Id*	NA	NA
[21]	*mcr-1.6*	Colistin, ampicillin, tetracycline, nalidixic acid, erythromycin, chloramphenicol, trimethoprim-sulfamethoxazole	*strA*, *aph(3*’*)-Ia*, *aph(4)-Ia*, *aac(3)-IVa*, *aac(6’)Ib-cr*, *strB*, *bla*_TEM-1B_, *bla*_OXA-1_, *oqxA*, *oqxB*, *floR*, *catB3*, *cmlA1*, *arr-3*, *sul1*, *sul2*, *sul3*, *tet*(B), *dfrA12*	NA	NA
[19]	*mcr-1*	Colistin, cefotaxime, cefepime, sulfamethoxazole/trimethoprim, ciprofloxacin	*aph(3*′*)-Ia/Ib, aadA1/A2, aac(6*′*)Ib-cr, aph(6)- Id, aph(4)-Ia, aac(3)-Iva, aph(3*′′*)-Ib, bla*_TEM-1B_, *bla*_CTX-M-14_, *bla*_OXA-1,_*oqxA, oqxB, qnrS2, aac(6*′*)-Ib-cr, fosA3, ARR-3, sul1, sul2, sul3, tet(A), tet(B*), *dfrA12*	NA	NA
[22]	*mcr-1*	Colistin, streptomycin, amoxicillin/clavulanic Acid, trimethoprim/sulfamethoxazole, tetracycline, chloramphenicol, florfenicol, nalidixic acid,	*strA, strB, sul2, tet*(B*), gyrA* (D87N), *phoP* (S131P), *phoQ* (T168A); *arnA* (L48Q, G51E, N184D, A195S, I247M, G284D, C303R, D337N, E554D, D631G), *arnC* (S320P), *arnD* (P164S, V216A); *pmrC* (I81V, Q415E), *pmrA* (S89T, R211G), *pmrB* (T18M, S76G, V86I, T114A); *pmrD* (L85S); *aadA1, aadA2, sul3, dfrA12, cmlA1*	*sinH, shdA, rpoS, mig-14, csgA, csgC, csgE, csgG, csgB, csgD, bcfD, bcfB, bcfC, bcfE, bcfF, bcfG, fimA, fimC, fur, ompD, lpfB, lpfC, lpfD, lpfE, misL, nlpI, ratB, pagN, stfA, stfC, stfD, stfE, stfF, stfG, safB, safD, safC, stbA, stbB, stbC, stbD, stcA, stcB, stcC, stcD, stdC, stdD, stdA, stdB, sthA, sthB, sthC, sthE, stiA, stiB, stiC, stiH, stjA, stjB, stjC, STM4575, fimI, fimD, fimH, fimW, fimY, fimZ, flgJ, flgB, flgC, flgF, flgG, cheA, cheB, cheR, cheW, cheZ, flgA, flgH, flgI, flgL, flgN, flhC, flhD, flhE, fliA, fliB, fliC, fliD, fliE, fliG, fliI, fliJ, fliL, fliM, fliN, flip, fliR, fliS, fliT, fliY, fliZ, flk, motA, motB, sodCI, siiE, mgtB, mgtC, iroB, iroC, iroD, iroE, iroN, sciS, STM0278, clpV, sciB, sciC, sciD, sciE, sciF, sciH, sciI, sciJ, sciK, sciL, sciM, sciN, sciO, sciQ, sciR, sciT, sciV, sciW, sseI, gogB, sseL, orgC, steA, hilA, invF, prgH, prgJ, avrA, hilC, hilD, iacP, iagB, invB, invC, invG, invJ, orgB, prgI, prgK, sicA, sicP, sipA, sipC, sipD, slrP, sopA, sopB, sopD, sopE2, spaO, spaR, sprB, sptP, pipB, pipB2, sifB, sopD2, sscA, sscB, sseK1, sseK2, ssrA, ssrB, steC, ssaI, sifA, spiC, ssaC, ssaD, ssaG, ssaJ, ssaK, ssaL, ssaM, ssaN, ssaP, ssaQ, ssaR, ssaU, sseA, sseB, sseE, sseJ, sspH2*	*arsC, arsB, arsA, arsR, arsD, cueO, pcoA, pcoB, pcoC, pcoD, fief, znuB, sitA, corC, corA, mgtA, merA, merR, merT, modC, modB, nikR, silP, silE, znuC, zraP, zupT, zur, terZ, terD, terC, terB, terE, terA, terW*
[18]	*mcr-1*	Colistin, ampicillin, tetracycline, nalidixic acid, chloramphenicol, cefotaxime, cefazolin, trimethoprim/sulfamethoxazole, cefuroxime, cefepime, gentamicin, ciprofloxacin, azithromycin, cefoxitin, ceftazidime, cefotaxime/clavulanic acid, ceftazidime/clavulanic acid, ceftazidime	*aac(6')-Iaa, bla*_TEM-1B_*, bla*_CTX-M-3_*, aph(6)-Id, aph(3'')-Ib, sul2, tet*(B)*, aac(3)-IId, aac(6')-Ib-cr, aadA2, bla*_DHA-1_*, bla*_OXA-1_*, qnrB4, mph(A), catB3, floR, ARR-3, sul1, tet*(A)*, dfrA12, bla*_CTX-M-55_*, aph(3')-Ia, aadA1, aph(4)-Ia, aac(3)-IV, bla*_CTX-M-14_*, oqxA, oqxB, fosA3, cmlA1, sul3, bla*_CTX-M-24_*, tet*(M*), bla*_DHA-1_*, aadA2b, bla*_CMY-2_*, qnrS2*	NA	NA
[25]	*mcr-1* and *mcr-3*	Colistin, aminoglycoside, β-Lactam, fluoroquinolone, sulfonamide, tetracyclines, florfenicol,	*aac(3)-IId*, *bla , strA, strB, sul2, tet*(A), *tet*(B), *bla*_CTX-M-55,_*qnrS1, catA2, floR*	NA	NA
[24]	*mcr-3.2*	Colistin, aztreonam, cefotaxime, ceftazidime, cefepime, chloramphenicol, gentamicin, kanamycin, trimethoprim/sulfamethoxazole, tetracycline	*sul3, tet*(A)*, tet*(B)*, aph(3¢)-Ic, aac(3)-IId, aadA1, aadA2, cmlA1, bla*_CTX-M-55,_*qnrS1*, *dfrA12*	NA	NA
[11]	*mcr-3*	Colistin, fluoroquinolone, trimethoprim, aminoglycosides, β-lactams, sulfonamides	*strA-strB*, *aph*(3*′*)-Ia, *bla*_TEM__-1b_, *tet*(A)-*tet*(B), *sul2*, *dfr*A5, *qnrS1, aac(6′)lb-cr*	NA	NA
[12]	*mcr-3.1*	Colistin, polymyxin, ampicillin, amoxicillin-clavulanic acid, ceftiofur, ticarcillin/clavulanic acid, nalidixic acid, ciprofloxacin, chloramphenicol, sulfisoxazole, tetracycline, minocycline, doxycycline, trimethoprim-sulfamethoxazole, gentamicin, tobramycin, streptomycin	*bla*_OXA-1_*, aac(6')Ib-cr, gyrA* (D87N)*, arr-3, catB3, cmlA1, sul1, sul2, sul3, tet(B), dfrA12, aph(6)-Id, aph(3'')-Ib, ant(3'')-Ia, aac(6')-Iaa, aac(6')-Ib, aph(4)-Ia, aadA8b, aadA3, aadA2, aadA1b, aadA1, strA, aac(3)-Iva, aph(3')-Ia*	NA	NA
[26]	*mcr-5*	Colistin, ampicillin; sulfamethoxazole; tetracycline; trimethoprim; chloramphenicol	*bla*_TEM-1B_*, sul2, sul1, tet*(A)*, tet*(B)*, dfrA1-like, dfrA5, dfrA12*	NA	NA
